# Nanotechnology-Driven Ultrasound/Photoacoustic Theranostics for Real-Time Imaging and Therapeutics

**DOI:** 10.7150/ntno.138452

**Published:** 2026-07-30

**Authors:** Praveen Kumar Annagowni, Aseem Setia, Madaswamy S. Muthu

**Affiliations:** 1 Product Development and Technical Services, Corepharma LLC 215 WoodAve, Middllesex, NJ 08846, USA.; 2 Department of Pharmaceutical Engineering and Technology, Indian Institute of Technology (BHU), Varanasi-221005, UP, India.

**Keywords:** ultrasound/photoacoustic imaging, endogenous and exogenous contrast agent, nanomedicine, theranostics

## Abstract

Theranostic technologies that incorporate nanotechnology into ultrasound (US) and photoacoustic imaging (PAI) show great promise for the diagnosis and treatment of a wide range of diseases. Despite recent advancements in nanomaterials, US/PA systems, which use both endogenous and exogenous contrast agents, are now more sensitive and effective in therapy. Intrinsic optical absorption-based label-free imaging is made possible by endogenous agents such as collagen, lipids, haemoglobin, and melanin. Exogenous contrast agents, on the other hand, are engineered to circumvent drawbacks such as low tissue selectivity and weak signal intensity. Advanced multimodal theranostic applications are made possible by these designed nanomaterials, which improve optical absorption, acoustic responsiveness, and targeted imaging. The benefits of optical high sensitivity and acoustic deep penetration are brought together in PAI, an integrated biomedical imaging modality. It may produce structural and functional pictures with enough contrast and resolution, providing a wealth of pathology data for disease-oriented diagnostics. Consequently, it has become an effective tool of precision nanomedicine and has discovered numerous uses so far. PAI is a hybrid imaging method that uses both optical and acoustic imaging to produce detailed pictures with a wealth of information about multiple parameters. This review explains the underlying principle of US/PAI bimodal imaging techniques, including their technical benefits, contrast agents, and biomedical applications. Additionally, we go over the difficulties and potential applications of US/PAI bimodal imaging in clinical settings.

## 1. Introduction

Biomedical imaging technology is an essential instrument in modern medicine 1. In order to improve overall health and treatment outcomes, it offers comprehensive biological information, helps with accurate disease diagnosis, directs treatment planning, and permits early detection of prospective health issues 2. Computed tomography (CT), magnetic resonance imaging (MRI), positron emission tomography (PET), optical imaging, and ultrasound imaging (US) are currently widely utilised clinical imaging modalities 3. High resolution, quick scanning, and multi-level imaging are all provided by CT. It does, however, have the disadvantage of radiation exposure and a limited capacity to distinguish between distinct tissues and lesions. Radiation-free imaging, multi-parametric imaging, superior soft tissue contrast, and high spatial resolution are some of MRI's best features 4. It offers comprehensive information about anatomy and function. But MRI's imaging sensitivity isn't always high, and it takes longer to scan and process picture data. It usually requires a lot of exogenous contrast agents, which raises the expense of equipment production and operation. PET is excellent at delivering molecular-level metabolic data and is utilised for tumour diagnosis, early illness detection, and assessment of treatment response 5. Its drawbacks include increased equipment expenses and the use of radioactive tracers during the imaging procedure, which involves radiation exposure concerns. Optical imaging produces images with excellent contrast and resolution. Through the detection of light's interaction with biological tissues, optical imaging offers exceptional spatial resolution and tissue contrast in a non-invasive manner. In the domains of cell biology, molecular biology, neuroscience, oncology, cardiovascular medicine, and drug discovery, it is extensively utilised in both clinical practice and research 6. Its imaging depth is nevertheless constrained by the photon propagation limit. The non-destructive viewing of deeper tissue features is made possible by ultrasound imaging, which uses echo signals from surfaces of variable impedance in the body 7. However, it can be difficult to see tissue structures with a diameter of less than 100 μm without ultrasound contrast agents (microbubbles). These substances may reduce sensitivity to pathological, physiological, inflammatory, and vascular alterations linked to illnesses while improving imaging capabilities. Additionally, ultrasound has trouble immediately obtaining functional data such as blood oxygenation 8. Ultrasound has significant advantages over other medical imaging methods, including its non-invasive nature, despite certain disadvantages, such as lower resolution and decreased efficacy in discriminating targets with modest acoustic impedance differences. It is crucial to remember that the ultrasonic waves central frequency affects the "deep penetration" feature of ultrasound imaging 9. Higher frequencies offer better resolution but shallower penetration, while lower frequencies enable deeper penetration at the expense of image resolution. In ultrasound imaging applications, this harmony between penetration depth and resolution is crucial. Due to these reasons, the combination of ultrasonic with new imaging technologies is becoming more popular as a means to attain non-destructive imaging that is both highly detailed and able to penetrate deeply 10. In this regard, photoacoustic imaging is showing promise as a novel imaging technology.

The photoacoustic (PA) effect, which permits energy transformation through the absorption of light and the resulting release of heat, is the basis of photoacoustic imaging (PAI) 11. PAI, which integrates the best features of optical and ultrasonic imaging (USI), can examine relatively deep tissues with pinpoint spatial precision and reveal molecular functional information. According to the PAI system, high-resolution imaging can be achieved with PAI, with resolutions ranging from tens of micrometres to hundreds of micrometres, and penetration depths exceeding the optical diffusion limit (~1 mm) and going up to several centimetres 12. Another useful application of PAI is the label-free evaluation of haemodynamics and physiologies, such as levels of haemoglobin oxygen saturation, which reflect the metabolic reaction of biological tissues. Unlike other modalities, PAI can acquire morphological vascular pictures and related sO2 information all at once thanks to haemoglobin, an endogenous contrast agent 13. Multispectral photoangiography utilises the unique optical absorption spectra of oxy- and deoxy-hemoglobin (HbO and HbR, respectively) to deliver sO2 distribution in the absence of injectable exogenous agents 14. Several biological investigations have proven that label-free sO2 analysis utilising PAI is feasible. When it comes to clinical applications involving humans, this technology has been extensively studied because injecting drugs can be somewhat difficult. Variations in tissue sO2 levels after medication delivery can be evaluated using PAI in this way. With the addition of therapeutic functionalities, contrast-enhanced PAI becomes capable of tracking medication delivery and treatment outcomes 15.

The optical signal can be absorbed by materials that are either produced within the body (endogenous) or created outside (exogenous) 16. Researchers have used peptide-based probes, gold nanoparticles, dyes, melanin, oxy-and deoxy-hemoglobin, and enzymes associated with cancer to identify tumours *in vivo*. Using non-ionizing laser power to stimulate specific tissues, PAI is able to provide real-time physiological data, such as blood oxygenation levels, and structural information about the area being studied. It is a cheap and non-invasive imaging method. Compared to traditional optical imaging, PAI provides a deeper penetration 17. Although PAI has numerous advantages, it does have some restrictions. The amplitude of the PA signal originating from deeper tissues and vessels can be reduced due to the depth-dependent nature of optical fluence, which is the deposited optical energy on the target region through laser. Consequently, PAI has limited usage in mainstream medical applications due to the difficulty in reaching the clinically necessary penetration depth. The inconsistency in acoustic characteristics across various tissue types is another problem that arises with PAI when imaging areas with overlapping tissue types. Furthermore, it is crucial to ensure that the biological system is compatible with and safe for use with any exogenous contrast agents utilised in PAI 18.

## 2. Basic Principle/Fundamentals of Ultrasound/Photoacoustic Imaging

**Figure 1:**
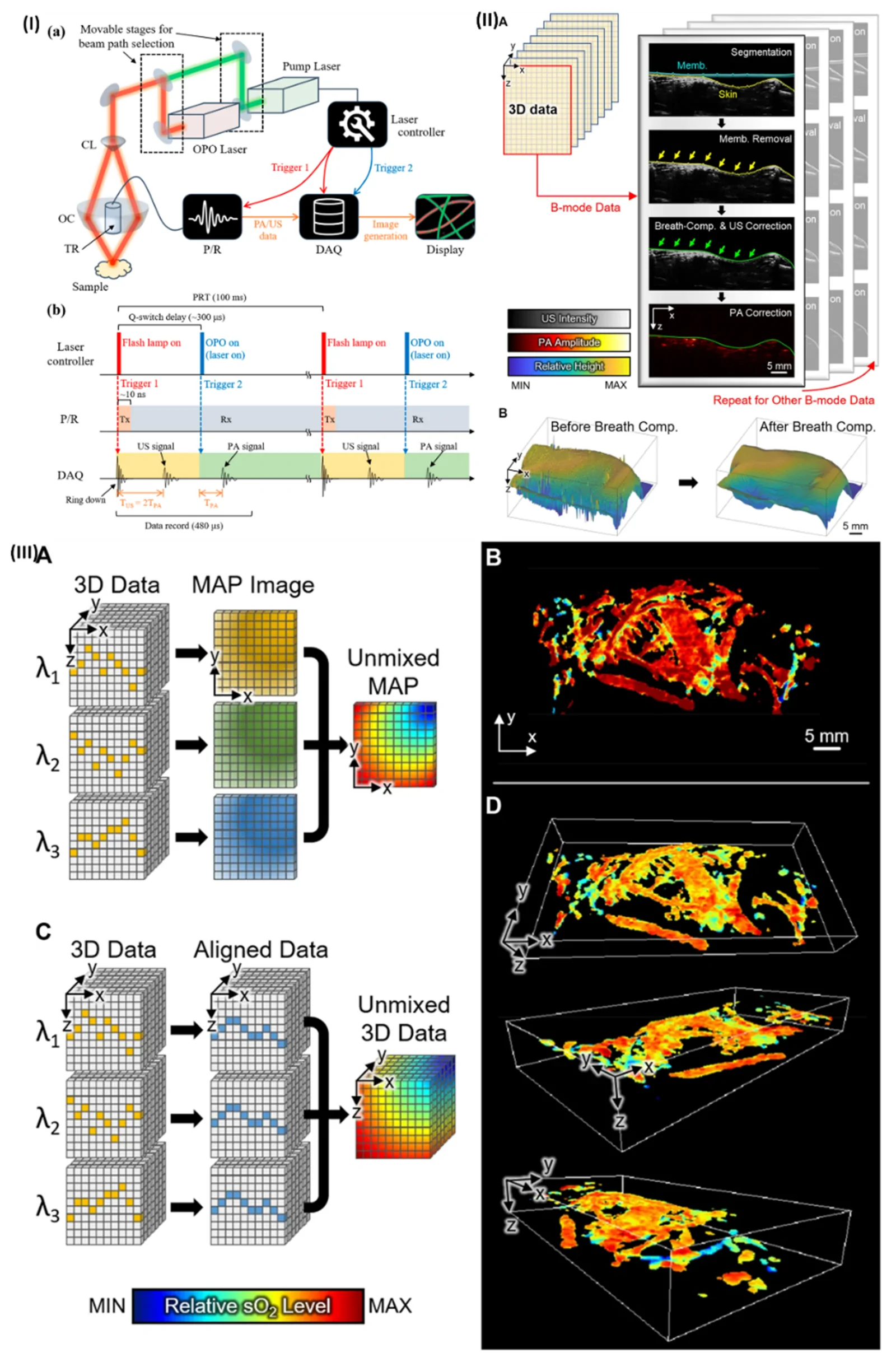
(I) Schematic representation of the imaging system that combines PA and US modalities. (a) System configuration. (b) Procedures for operating the system that allow for dual-modal imaging. Reproduced with permission from 26. (MDPI©2021) (II) (A) A schematic depicting the US-guided breath-compensation technique. (B) Comparison of the skin's surface profile. (III) Relative sO2 distribution in mice. Reproduced with permission from 25. (Frontiers©2022).

A relatively new method of diagnostic imaging, photoacoustics, allows for the acquisition of tomographic information about tissues or lesions in three dimensions. Localised heating and energy absorption by photosensitive substances, brought about by pulsed laser irradiation, lead to absorber expansion 19. The PA effect, which occurs when the surrounding tissues are compressed due to the expansion, is responsible for sending acoustic waves to the transducer for use in picture reconstruction **(Figure 1 (I))**. Varying structures of the test object can be distinguished by using the PA effect, which is based on the difference in acoustic waves induced by varying light absorption rates in various species 19. The PA effect, in which light is converted into sound waves, is the fundamental premise of photoacoustic imaging. According to their optical absorption properties, optical chromophores in biological tissues absorb light energy when exposed to brief pulsed light, usually with a pulse width of a few nanoseconds 20. The absorbed light energy is then released in two ways: first, as a shifted-wavelength emission of light (the principle of fluorescence); and second, as a release of heat (the result of thermoelastic expansion). Acoustic waves called PA waves are produced when the optically absorbing chromophores contract back to their original volume due to the short pulse length, which causes the thermoelastic expansion to dissipate quickly. These principles form the basis of PAI, which allows the visualisation of biological tissues optical absorption properties 21. As a result, it can give molecular functional information that is comparable to that which is received through pure optical imaging. The contrast of PA pictures can be improved by exogenous agents as well as endogenous chromophores, such as oxy- and deoxy-hemoglobins, melanin, and lipid 22. Both preclinical investigations in small animals and clinical studies in humans have made use of PAI to visualise the biological distribution of optical absorption properties. In PAI, standard transducers and data-collecting systems from the United States can be used to capture the PA waves that are generated. Consequently, a single imaging platform can easily incorporate PA and US pictures. **Figure 1 (II)** shows that the structural information from USI and the molecular functional information from PAI are complementary, but that their underlying information is distinct. Consequently, PAUSI is important because, compared with combining two imaging modalities, it can acquire more useful information for investigating biological systems 23. A data acquisition (DAQ) system, detectors, and an optical source are the usual components of a PAI system. To maximise light delivery to the target tissue, the optical source, usually a pulsed laser, provides excitation and can have its beam shape optimised by lenses or diffusers. After that, various detectors, including optical sensors, piezoelectric micromachined ultrasonic transducers, or traditional piezoelectric ultrasound transducers, are chosen according to individual needs in order to pick up the produced PA signals. After passing through the DAQ system, these electrical impulses are transformed into pictures that can be used 24. Image reconstruction employing methods like delay-and-sum, filtered back-projection, or time-reversal follows signal digitisation, filtering, demodulation, and amplification. Based on the application's needs, PAI configurations can be customised to include additional signal and image processing tools, preamplifiers, and multiplexer boards.

In a study, Lee *et al*. developed an ultrasound-assisted respiratory motion correction technique for three-dimensional photoacoustic macroscopy. After collecting *in vivo* photoacoustic and ultrasound data across the entire mouse body, they used the ultrasound data to correct for skin-induced distortions. Next, the relevant photoacoustic data were adjusted for motion using the derived distortion parameters. When applied to multispectral photoacoustic data, the compensatory method allowed for the visualisation of three-dimensional haemoglobin oxygen saturation throughout the entire body of living mice. The three-dimensional pictures that came out of it prove that the new technique has many potential applications in the biomedical field, such as tracking medication administration, imaging cancers, and studying blood vessel networks **(Figure 1 (III))** 25.

## 3. Contrast agent for Photoacoustic Imaging

One of the most promising new directions in imaging technology development recently is the use of PA imaging for monitoring biological tissues in living organisms 27. The lack of highly efficient contrast agents severely limited PA imaging's further clinical applicability, despite the method's promise as a biomedical imaging tool 28. Haemoglobin, chromophores, and lipids are only a few examples of endogenous contrast agents that, when exposed to light, can transform it into pulsed adenosine signals that convey unique information. There is a lot of background noise in tissues when using endogenous contrast agents, and they can't penetrate very deep, so one can't use them to choose where to do the imaging. Thrillingly, imaging technology and scientific instrument advancements in the last few years have made it possible to use a plethora of exogenous contrast agents to produce sufficient PA signals *in vivo* or in particular pathological tissues, thereby revealing disease pathology 29. When doing PAI, contrast agents, both endogenous and exogenous, are utilised. Melanin and haemoglobin are two examples of endogenous substances that create strong PA contrast signals because they absorb more light in the visible and near-infrared (NIR) spectra than nearby tissues. Nevertheless, as endogenous contrast agents do not provide sufficient information when imaging a tumour in its early stages, biocompatible exogenous contrast agents are utilised. By comparison to endogenous contrast agents, the latter improves picture quality and increases signal contrast 30.

### 3.1. Endogenous contrast agent for photoacoustic imaging

In biological tissues, substances such as haemoglobin, melanin, lipids, collagen, bilirubin, water, DNA, and RNA serve as endogenous contrast agents since they are intrinsic chromophores. The tumour microenvironment contains metabolites and intracellular factors that control tumour metabolism and behaviour 31. The utilisation of endogenous contrast agents in tumour photoacoustic molecular imaging is based on the fact that various molecules produce different photoacoustic signals, and that these differences arise at the molecular level as tumours evolve 32. Endogenous contrast agent imaging has several advantages over conventional methods, including less potential for drug exposure, more accurate representation of tissue structure and function, and increased biological safety. Having said that, endogenous contrast agents do not have a very targeted distribution. Except for water, most endogenous contrast agents have a greatly reduced extinction coefficient in the near-infrared (NIR) window. Additionally, the inherent contrast is not always enough for imaging, which can limit the detection range of photoacoustic imaging technology. It is necessary to use exogenous contrast agents in order to enhance contrast and target. To visualise physiological responses in PAI, contrast agents can be endogenous light-absorbing chromophores like oxy-hemoglobin, deoxy-hemoglobin, melanin, and lipids. The bladder, lymphatic system, tumours, and other optically transparent organs are the only ones that can produce endogenous chromophores. Additionally, all PA signals are usually dominated by two types of haemoglobin 33. Hence, there has been a plethora of research into methods for improving contrast by means of various exogenous agents, such as organic dyes, carbon nanostructures, gold nanoparticles, semiconducting nanoparticles, and fluorescent proteins.

**Figure 2:**
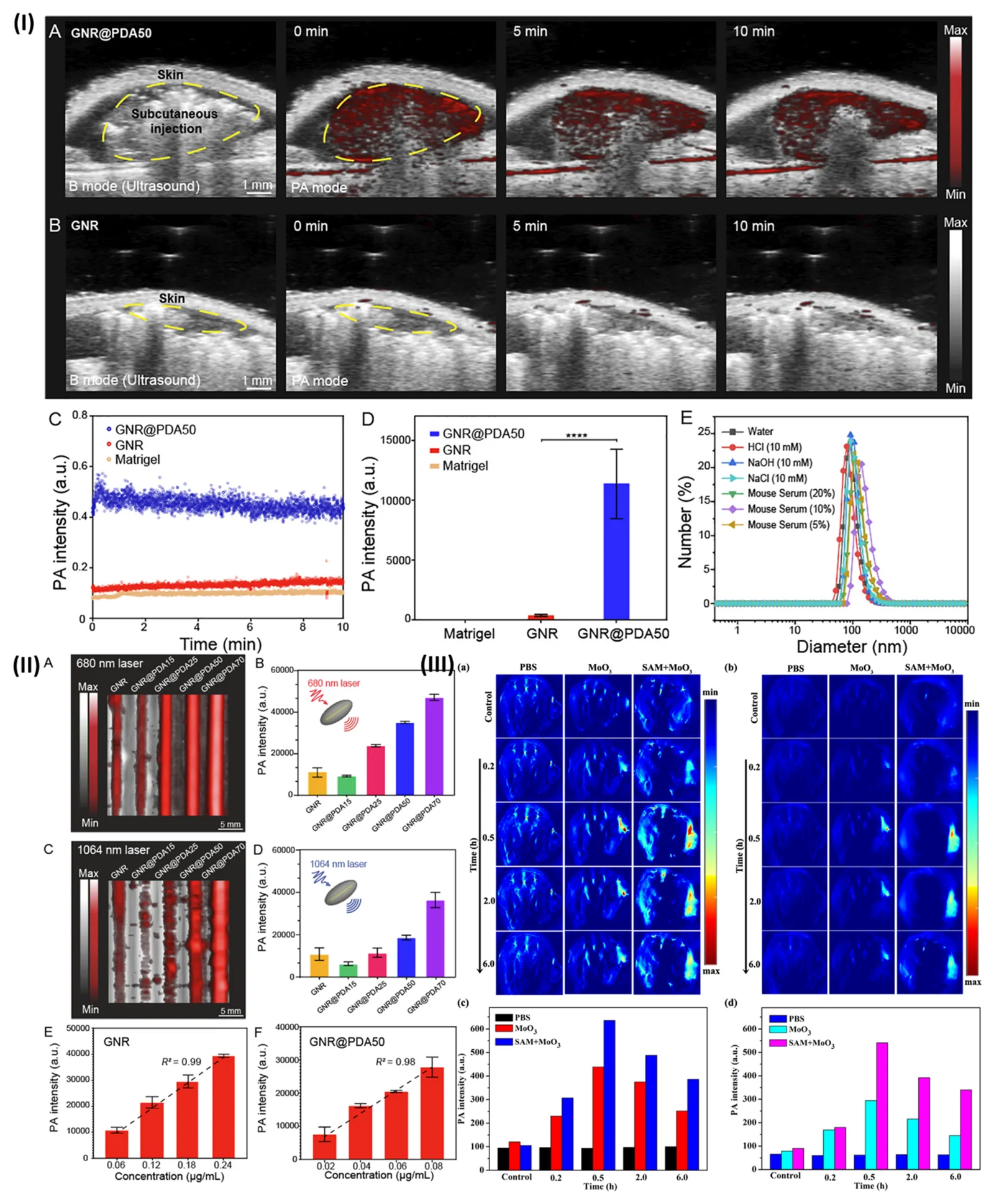
(I) PA imaging of GNR@PDA *in vivo*. (II) PA performance of GNR and GNR@PDA. Reproduced with permission from 34. (ACSPublications©2021). (III) Colon cancer microenvironment-activated PA images. Reproduced with permission from 35. (ACSPublications©2021).

Yim *et al*. described the fabrication of monodispersed GNR-melanin nanohybrids featuring a uniformly deposited and tunable polydopamine (PDA) shell surrounding the gold nanorods. Greater optical absorption, cross-sectional area, and thermal confinement in GNR@PDAs resulted in a PA signal that was three times stronger than in pure GNRs. Crucially, GNRs showed a considerable attenuation of 77% during the 5-minute laser illumination in the NIR-II window, but GNR@PDAs only showed a 29% decrease in PA signal. The GNR@PDAs were able to retain 87% of their initial PA signal *in vivo* following only 10 minutes of laser illumination. This PDA-enabled approach opens up new possibilities for photothermal and photodynamic regimens, among other photomediated biomedicines, and allows for a more reasonable construction of strong PA imaging probes **Figure 2(I)&(II)** 34.

Moreover, Wang *et al*. synthesized water-dispersible MoO3 nanoparticles and demonstrated that endogenous H_2_S could selectively trigger NIR-II-mediated photoacoustic imaging and photothermal therapy in colon cancer tissues. So, to conduct PA imaging and PTT on colon cancer tissue, MoO3 nanoparticles were utilised as an NIR-II nanoprobe. In addition, the NIR-II window demonstrated effective photothermal efficiency, reduced background interference during PA imaging of living tissue, and a good PTT effect for colon cancer, as compared to NIR-I. This study provided a method for the detection and therapy of colon cancer by creating a tailored nanoprobe for near-infrared (NIR-II) PA imaging of bioactive small compounds **Figure 2(III)** 35.

### 3.2. Exogenous contrast agent for photoacoustic imaging

Even while endogenous chromophores can be used for imaging, they can interfere with the background signal when contrast agents are used 36. To differentiate between the tissue's photoacoustic signal and the introduced signal, a contrast agent usually needs a substance with a high molar extinction coefficient and peak absorption in the near-infrared bandwidth 37. The ideal exogenous contrast agent would also have excellent photostability, little quantum yield, minimal toxicity and immunogenicity, excellent biocompatibility, and excellent target affinity and specificity 38. There are three main components to think about when making exogenous contrast agents: the material that can provide photoacoustic signals, the part that can identify specific markers or biological activities, and how to put it all together 39.

#### 3.2.1. Dyes based contrast agent for photoacoustic imaging

Indocyanine Green (ICG), IRDye800CW, AlexaFluor750, methylene blue, and a plethora of other biocompatible dyes absorb in the optical window; all have found usage in PA imaging. The renal system is able to swiftly remove these dyes because they are usually very small molecules, measuring just around 1 nm. For the most part, these dyes are reserved for optical imaging, and a few of them are fluorescent. The absorption spectra are the sole tool used in PA imaging. Since more of the absorbed energy is transformed into the photoacoustic signal rather than being radiated, a low quantum yield makes PA signal creation with fluorescent dyes more efficient 40.

**Figure 3:**
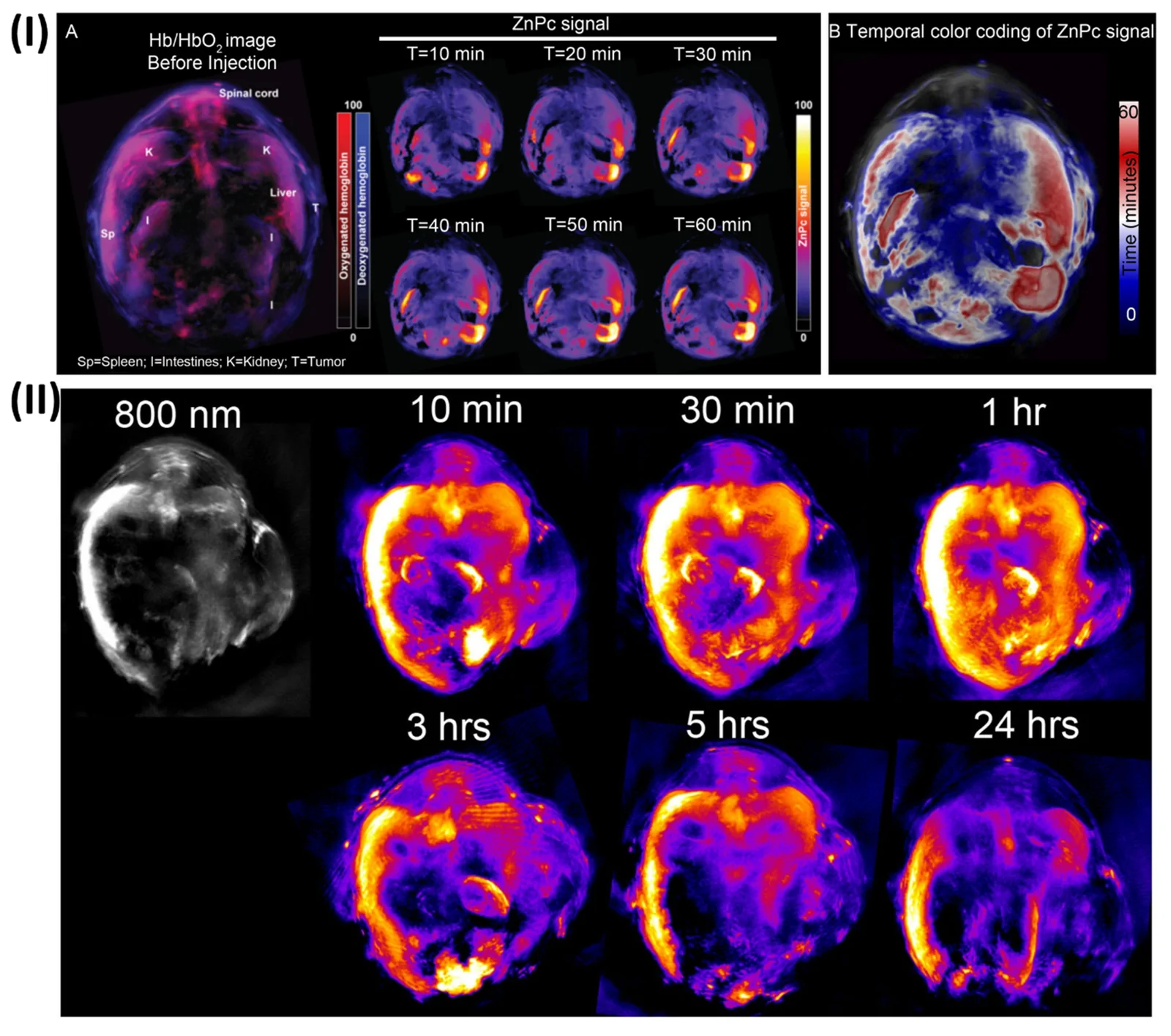
** (I)** (A) Images of transverse slices taken by an *in vivo* MIP camera before and one hour after an injection reveal the slow buildup of probes within the tumour location and other organs. (B) Colour-coded time-resolved MSOT images taken one hour after injection show the probe's peak localisation at the tumour location and other organs at that time. **(II)** Strong probe MSOT signals within the tumour site, liver, spleen, kidneys, and intestines were demonstrated in *in vivo* non-background-corrected MIP images of transverse slices through mice at different time periods post-injection. Reproduced with permission from 41. (Scientific reports©2014).

Ho *et al*., examined the photoacoustic activity of multiple photosensitizers. Afterwards, they used photoacoustic imaging to assess its tumour localisation effectiveness and biodistribution in a mouse model at various time periods. They discovered that the probe localised to the tumour within 10 minutes of injection, achieved peak concentration at roughly 1 hour, and was eliminated within 24 hours, suggesting that photosensitisers have potential as contrast agents in photoacoustic imaging *in vivo*. Cancer progression and treatment outcomes can be monitored *in vivo* using a combination of photosensitizer-recognized advantages, such as tumour uptake and photodynamic therapy efficacy, and photoacoustic imaging **(Figure 3)** 41.

Moreover, Chaudhary *et al*., investigated how the photo-stability, loading, release, and photoacoustic signal strength of ICG, are affected by the chemical and physical surface functionalisation of mesoporous silica nanoparticles (MSNs). To execute chemical functionalisation, MSNs were modified with silanes that included amine (NH2) or phosphonate (PO3) terminal groups. To execute physical changes, lipid bilayer (LB) or layer-by-layer (LBL) polyelectrolyte coatings were applied to the ICG-loaded MSNs. When comparing NH2-MSNs with PO3-MSNs, they found that the former has a far higher ICG mass loading capacity (16.5 wt%) and a slower release of ICG (5% in PBS over 48 hours), respectively. After 48 hours of vacuum loading, the physically changed MSNs (LBLMSNs and LBMSNs) released less than 10% ICG, with a loading percentage of about 9 weight percent. While functionalised MSNs containing ICG did not photo-degrade, pure ICG was very photo-unstable and lost 20% of its photoluminescence (PL) after 3 hours of exposure to 800 nm. Out of all the formulations that were examined, NH2-MSNs and LBLMSNs showed an increase of four times the strength of the *in vitro* PA signal at an equivalent ICG dose of 200 μg mL-1. When administered subcutaneously into mouse cadavers, NH2-MSNs and LBLMSNs outperformed pure ICG by 1.29 and 1.43 fold, respectively, in PA imaging, mirroring results shown in *in vitro* studies 42.

#### 3.2.3. Organic contrast agent for photoacoustic imaging

One common option for PA contrast agents is organic dyes, which are widely available in the market. Imaging at depth becomes a major challenge when using dyes and dye-conjugates for PA imaging since their molar extinction coefficient is four to six orders of magnitude lower than that of gold-based contrast agents 43. The absorbance maxima of most commonly used dyes are also in the 600-800 nm region, making SO_2_ quantification a challenge. There has been a lot of interest in organic PA contrast agents such porphyrins, cyanine-based dyes, melanin, and perylene-diimide (PDI) because of their great biodegradability and biocompatibility 44.

Lovell *et al*. developed self-assembled porphyrin-based nanovesicles that demonstrated remarkably high and tunable extinction coefficients along with structure-dependent fluorescence self-quenching, enabling enhanced photothermal conversion and photoacoustic imaging performance. Porphysomes made it possible to use photoacoustic tomography to sensitively see lymphatic systems. Low-background fluorescence imaging may become possible if dissociation could restore near-infrared fluorescence production. Mice administered 1,000 mgkg-1 of porphysomes intravenously showed less acute toxicity due to the organic compounds' enzymatic biodegradability. Porphysomes, like liposomes, have a big watery core that can be loaded passively or aggressively. Porphysomes accumulated in xenograft tumours after systemic treatment, and photothermal tumour ablation was caused by laser irradiation. Porphysomes' biocompatibility and optical characteristics show that organic nanoparticles have multimodal imaging and therapeutic potential 45. Wu *et al*. created GBM tumor-targeted nanoparticles called TCFNP@iRGD. This study evaluates their potential in detecting glioma-derived cells intracranial xenograft models using PA imaging for the first time. The outstanding tumor-specific PA mapping performance of TCFNP@iRGD demonstrates its amazing potential in the clinical diagnosis of GBM 46.

**Figure 4:**
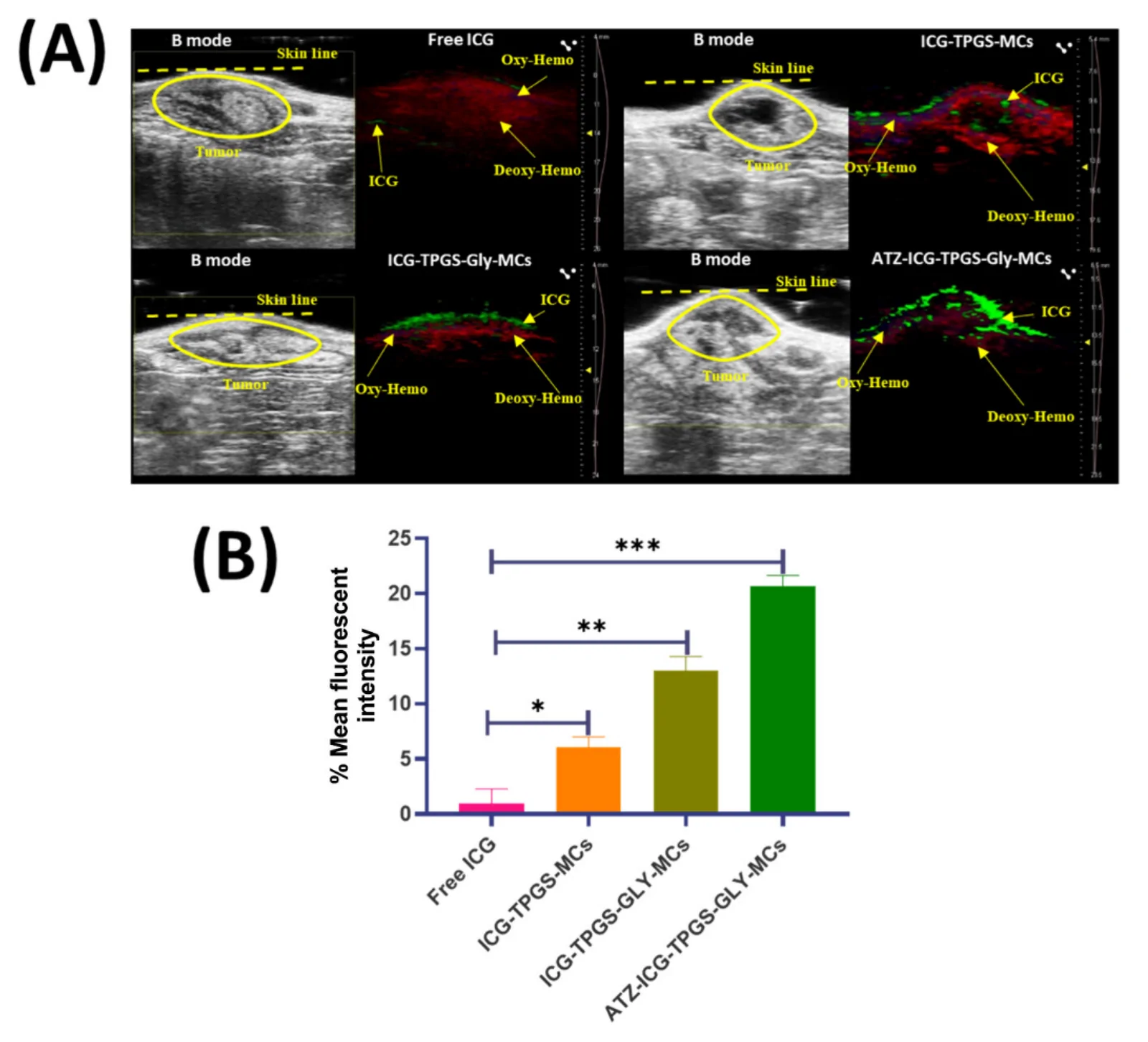
(A) Ultrasound/photoacoustic imaging was used to assess the distribution and delivery of free ICG and ICG-loaded MCs to the breast tumour. (B)The average green signal fluorescence intensity of both ICG-loaded and free MCs. Reproduced with permission from 48 (ACSPublications©2025).

Setia *et al*., developed methylene blue-loaded targeted and non-targeted mixed micelles for the benzopyrene-induced lung tumor model. It is highly probable that the tailored formulation improves the retention and localisation of mixed micelles by increasing their binding to lung tissue receptors. Based on these findings, mixed micelles appear to be a good option for photoacoustic imaging 47. Furthermore, Randhave *et al*., developed ICG and ICG-loaded micelles for the DMBA-induced breast tumor model. Study results suggest that targeted formulation increases tumor-specific green photoacoustic signals, which in turn increases tumor-specific ICG concentration in photoacoustic imaging, leading to better visualisation and perhaps more effective therapy** (Figure 4)** 48.

#### 3.2.3. Inorganic contrast agent for photoacoustic imaging

Inorganic contrast agents have emerged as very useful materials for photoacoustic imaging due to their superior optical absorption, photothermal conversion efficiency, and variable physicochemical features. Metallic nanoparticles such as gold, silver, copper sulphide, iron oxide, and bismuth-based nanostructures are intensively researched for increasing photoacoustic signal production 49. These agents improve imaging sensitivity, spatial resolution, and deep tissue visualisation by efficiently converting absorbed near-infrared light into ultrasonic vibrations. Additionally, inorganic nanoparticles can be functionalised with targeted ligands, medicines, or polymers for theranostic purposes. They are capable of doing biomedical imaging biomedical imaging imaging biomedical research 44.

Biocompatible gold nanostars with an adjustable geometry are synthesised using a new, surfactant-free process by Yuan *et al*. This allows for *in vivo* imaging by tuning the plasmon band into the near-infrared region, specifically the tissue diagnostic window. The absorption spectra produced by the theoretical modelling of multiple-branched 3D nanostars were in excellent agreement with the experimental data. The plasmon band intensity grows as the star's branch count, branch length, and total size increase, and its shift was found to be caused by changes in the branch aspect ratio. An extraordinarily robust two-photon photoluminescence (TPL) mechanism was observed in nanostars. Researchers in the lab found that gold nanostars can be a useful contrast agent for biological imaging by using transperitoneal magnetic resonance imaging (TPL) to study wheat-germ agglutinin (WGA) functionalised nanostars on BT549 breast cancer cells and PEGylated nanostars circulating in the vasculature 50.

Chen *et al*. documented the production of extremely miniature gold nanorods with an aspect ratio that are 5-11 times smaller than their regular-sized counterparts, and which absorb in the near-infrared-II spectrum. Small nanorods, when illuminated with nanosecond pulsed laser light, provide a photoacoustic signal that is three times more stable in temperature and three times stronger than larger absorption-matched nanorods. Their findings, which were surprising, are confirmed by a combination of theoretical and numerical analyses, which show that the photoacoustic signal is proportional to the nanoparticles' optical absorption and surface-to-volume ratio. The utilisation of these small targeted nanorods results in 4.5 times greater photoacoustic contrast and a 30% improvement in agent delivery efficiency in tumor-bearing mice 51. Combining functionalised black-titanium nanoparticles with iridium complexes and cancer cell membranes, Shen *et al*. offer a nanoplatform for hierarchical targeted synergistic photothermal and sonodynamic cancer imaging and therapy. The particles demonstrated effective heat generation and catalytic formation of reactive oxygen species in response to irradiation and ultrasound, respectively. A hierarchical targeting technique was shown by the nanoparticle formulation, which selectively localised in the mitochondria and accumulated preferentially in diseased cells over non-cancerous ones, as well as in the tumour within a mouse model. In a mouse model, the nanoparticles served as both an imaging agent and a therapeutic agent, eradicating a tumour when exposed to a combination of ultrasonic and synergistic irradiation at 1064 nm 52.

### 3.3. Stimuli-responsive contrast agent for photoacoustic imaging

Modulating the spectrum properties of stimuli-responsive molecules in real-time enhances the functional and chemical specificity of PAI. The first category includes devices that can be activated from the outside, allowing for non-invasive, user-controlled on/off modulation 53. The second category includes sensors that can be activated from within, acting as molecular probes to reveal biochemical signatures within living systems. These sensors are comparable to activatable fluorescent dyes used in histological assays 54. To combat cancer, photodynamic therapy (PDT) uses a combination of photoinduced ROS to induce direct oxidative stress and immunogenic cell death to stimulate immunological responses. Strong optical absorption and possibly partial fluorescence (FL) or photoluminescence are characteristics of the administered photosensitizers. Unfortunately, there are some drawbacks to traditional PDT. One of them is the possibility of harming normal tissues. Another is that the PA and FL modalities do not provide the best imaging contrast due to competing energy routes 55.

In a study, Gao *et al*. created a smart one-for-all nanoagent whose photophysical energy transformation processes may be easily controlled by external light stimuli. The two light-switchable versions of a dithienylethene-based molecule make it an attractive building block for synthesis. The majority of the absorbed energy for photoacoustic (PA) imaging dissipates in the ring-closed state due to nonradiative thermal deactivation. The molecule's ring-open conformation is ideal for photodynamic treatment and fluorescence because of its aggregation-induced emission qualities. Fluorescence imaging during surgery can detect even the tiniest remaining tumours with excellent sensitivity, and *in vivo* studies show that preoperative PA and imaging aid in high-contrast tumour delineation. Additionally, the nanoagent has the ability to trigger immunogenic cell death, which in turn triggers antitumor immunity and effectively suppresses solid tumours. The smart one-for-all agent developed in this work has the potential for multifunctional biomedical applications due to its ability to optimise photophysical energy transformation and related phototheranostic features through a light-driven structural switch 56.

Furthermore, Deng *et al*. introduced VPH-5DF, a mitochondria-targeting near-infrared (NIR) fluorescent probe that can measure polarity, viscosity, and H_2_O_2_ through two separate NIR channels. The probe demonstrates a strong sensitivity to the microenvironmental viscosity/polarity in the deep NIR channel and can particularly detect H_2_O_2_ by NIR emission. In addition to tracking mitochondrial polarity, viscosity, and changes in endogenous/exogenous H_2_O_2_ levels, the probe uses a battery of metrics to differentiate cancer cells from healthy cells. It is also possible to use VPH-5DF to track changes in polarity, viscosity, and H_2_O_2_ levels in live cells as they undergo drug-induced pyroptosis. Xenograft tumour models showed that after VPH-5DF treatment, a strong antitumor response was elicited by chemotherapy-induced oxidative damage to the mitochondria in tumour cells. Anticancer treatment and accurate *in vivo* cancer diagnostics are both made possible by this triple-response theranostic prodrug 57.

## 4. Nanotechnology-Enabled Ultrasound/Photoacoustic Theranostics

There are a variety of formulation strategies that have been made possible by nanotechnology; these include wound-healing nanosystems, thrombolytic nanoplatforms, polymeric micelles, and ultrasound (US) and photoacoustic imaging (PAI)-guided theranostics 58. The controlled release capabilities, high drug-loading efficiency, biodegradability, and biocompatibility of polymeric nanoparticles make them an ideal candidate for the co-delivery of imaging contrast materials and therapeutic medicines. To improve solubility, circulation time, and tumor-specific accumulation, hydrophobic medications and photoacoustic probes can be encapsulated in polymeric micelles made of self-assembled amphiphilic copolymers 59. In contrast, thrombolytic nanoplatforms aim to enhance vascular disease diagnosis and treatment by integrating imaging functionalities with thrombolytic agents. This allows for targeted drug delivery, ultrasound-triggered clot dissolution, and real-time visualisation of thrombus formation, all while reducing systemic adverse effects. Nanoplatforms for wound healing combine imaging capabilities with therapeutic compounds, antibacterial agents, or growth factors to expedite tissue regeneration and provide non-invasive, continuous monitoring of the healing process 60. These nanoplatforms have several biological uses, but when combined they allow for more precise imaging, more controlled and stimulus-responsive drug release, better therapeutic precision, and the ability to diagnose and treat patients at the same time. By combining US and PAI with these multifunctional nanosystems, theranostic techniques have been greatly enhanced, allowing for better visualisation of diseases, monitoring of treatments, and personalised therapeutic interventions in the fields of regenerative medicine, cardiovascular disorders, and cancer 61.

### 4.1. Polymeric nanoparticles for breast cancer therapy

Nanoparticles composed of polymers, either naturally occurring or man-made, can have diameters between one millimetre and one thousand nanometres 62. These nanoparticulate structures are designed to contain drugs, genetic material, or materials utilised in medical imaging 63. In the realm of biomedicine, they serve a variety of purposes, such as helping with diagnoses, facilitating imaging operations, and delivering drugs 64. Polymeric nanoparticles made from biocompatible materials can be administered *in vivo* without the risk of toxicity or immunological responses 65. Biocompatible polymers that are frequently utilised include chitosan, gelatin, polyethylene glycol (PEG), and poly(lactic-co-glycolic acid) (PLGA). Additionally, polymeric nanoparticles offer a versatile platform for encasing hydrophilic and hydrophobic medications within their polymeric matrix 66. The medication is protected from deterioration by this encapsulation procedure, which also improves its solubility, stability, and bioavailability. Pharmaceutical release from polymeric nanoparticles can be tailored and controlled by adjusting the polymer composition, nanoparticle size, and surface characteristics 67, 68. This mechanism-controlled release kinetics enable the gradual and prolonged release of medications, which reduces the frequency of administration and the likelihood of side effects. Furthermore, the precise delivery of medications to targeted tissues, including tumours, is made possible by the attachment of targeting ligands to polymeric nanoparticles 69. Notably, precision distribution reduces total exposure and unwanted side effects while increasing medicine concentration at the targeted site. Additionally, polymeric nanoparticles shield drugs or genetic material contained inside them from enzyme degradation and immune system clearance 70. Longer circulation times and improved stability result from this. Imaging contrast agents like fluorescent dyes, magnetic nanoparticles, or radioactive tracers are examples of polymeric nanoparticles. This makes it possible to image tissues or disease biomarkers non-invasively. To enable specific molecular imaging applications, they can also be altered with targeting agents. Notably, certain polymeric nanoparticles integrate therapeutic and diagnostic properties within a single nanoparticle system to generate theranostic platforms 71, 72. They support the customisation of therapeutic options by continuously monitoring medication distribution, pharmacokinetics, and treatment response 73. Biodegradable polymeric nanoparticles break down into innocuous compounds within the body, which facilitates their removal and reduces the possibility of long-term accumulation or toxicity 74, 75.

**Figure 5:**
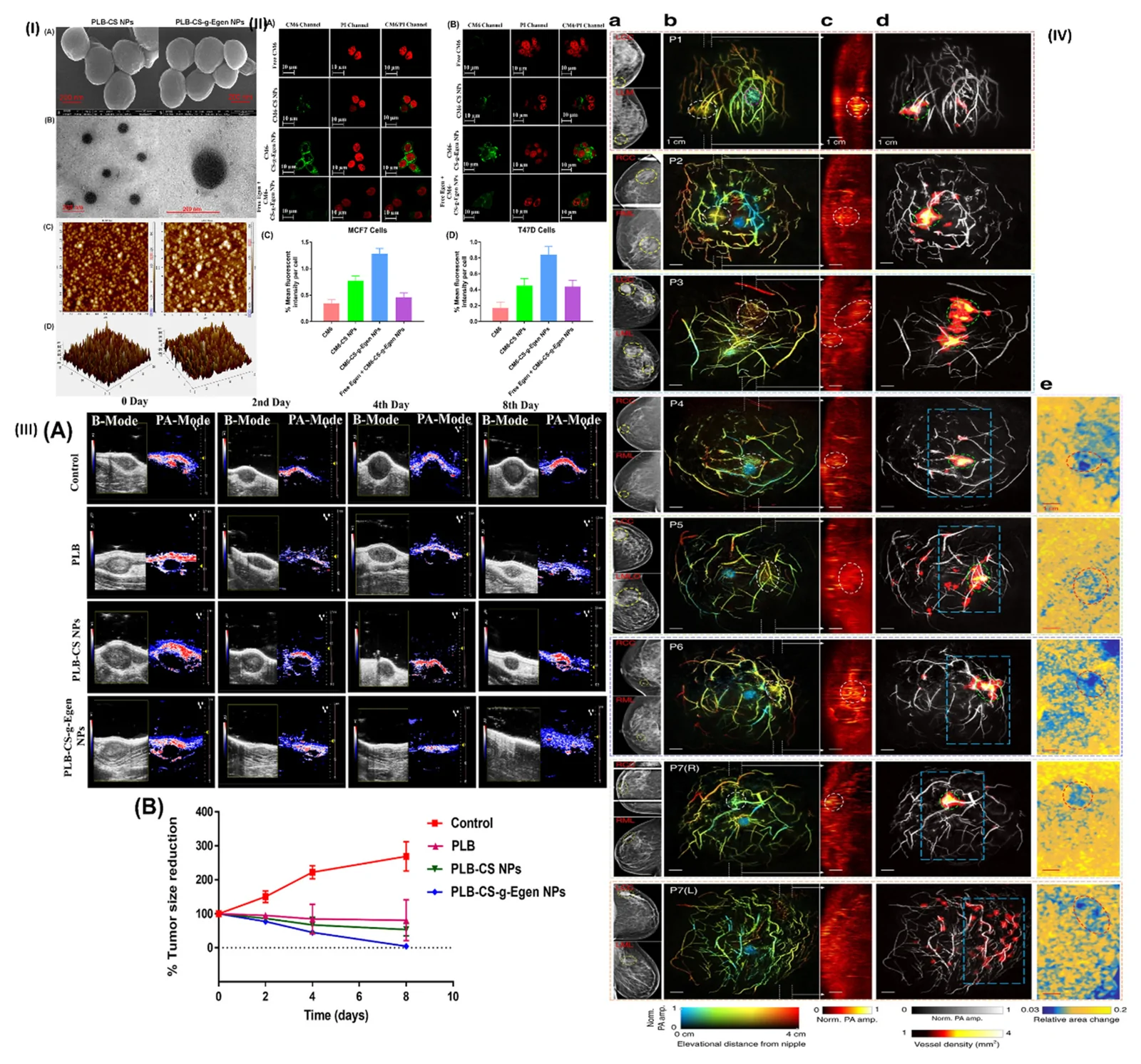
(I) Morphology of the nanoformulations; (II) Intracellular tracking of nanoformulations in (A) MCF7 cells and (B) T47D cells; (III) imaging of the breast tumour using USG/PAI methods both prior to and following NPs therapy. Reproduced with permission from 76. (ACSPublications©2023). (IV) SBH-PACT of cancerous breasts. Reproduced with permission from 77. (NatureCommunications©2018).

A polymeric nanocarrier based on chitosan and grafted with oestrone (Egen) was prepared by Mehata *et al*. to deliver palbociclib (PLB) specifically to breast cancer. These NPs were made by ionic gelation-based solvent evaporation and then studied for apoptosis, drug entrapment efficiency, surface shape, surface chemistry, cellular uptake, cytotoxicity, and zeta potential. Using oestrogen receptor (ER)-expressing MCF7 cells and T47D cells, an *in vitro* cytotoxicity experiment was conducted. The results indicated that the targeted NPs were more cytotoxic than other formulations. Pharmacokinetic experiments conducted in living organisms showed that the half-life and bioavailability were enhanced by a factor of 2-3 when the PLB was trapped in the NPs. Additionally, USG/PA imaging demonstrated that targeted NPs eradicated the tumour, decreased the volume of hypoxic tumours, and inhibited tumour angiogenesis more effectively. Studies on histopathology and *in vitro* hemocompatibility further supported the idea that NPs were safe to utilise in clinical settings and biocompatible **(Figure 5(I-III)** 76. Moreover, A technique for revealing detailed angiographic features in human breasts was developed by Lin *et al*. and named single-breath-hold photoacoustic computed tomography (SBH-PACT). The SBH-PACT boasts a high spatial and temporal resolution of 255 µm in-plane and a 10 Hz 2D frame rate, in addition to a deep penetration depth of 4 cm *in vivo*. A volumetric image can be obtained by scanning the entire breast in just one breath hold (~15 s) and then using 3D back-projection to rebuild it with almost no breathing-induced motion artefacts. With its excellent spatial resolution and ability to detect tumours by greater blood vessel densities, SBH-PACT shows early promise for very sensitive imaging of radiographically dense breasts. Dynamic investigations, like photoacoustic elastography, which detects tumours by displaying decreased compliance, are made possible by the fast imaging speed, which also allows for blood vessel imaging. They took pictures of breast cancer patients with a wide range of skin tones and cup sizes (B to DD). With no need for ionising radiation or external contrast, SBH-PACT safely detected all of the tumours **(Figure 5 (IV))** 77.

Randhave *et al*., synthesized TPGS-Glycogen (TPGS-GLY) for breast cancer therapy. To target hypoxic breast tumours, they utilised the solvent casting approach to make polymeric nanomicelles (MCs) encapsulated with palbociclib (PLB). The surface of these MCs was then coated with an anti-programmed cell death-ligand 1 (PD-L1) antibody. Various physicochemical analyses have been carried out. Storage, salt ion, and serum stability tests all showed that the MCs remained unchanged. The drug release patterns *in vitro* at two different pH levels (5.5 and 7.4) show that the release of drugs within cells is driven by the endosomal pH. Compared to pure PLB, the cytotoxicity of the targeted MCs was 30.94 times higher in MCF-7 cells and 115.9 times higher in MDA-MB-231 cells. Each of the MCs that were generated has been subjected to research on cellular absorption, apoptosis, and reactive oxygen species. Targeted MCs not only eradicated the tumour but also reduced hypoxic tumour volume and inhibited tumour angiogenesis, according to USG/PAI in rats with DMBA-induced breast cancer. Targeted micelles have improved tumour localisation, according to an evaluation of MCs targeting efficiency toward breast tumours utilising USG/PAI imaging with the clinical dye indocyanine green (ICG). Additionally, DiD dye has been used to study the biodistribution of organs using intravascular ultrasound (IVUS) imaging 48.

### 4.2. Micelles for lung cancer therapy

Micelles are colloidal particles that typically range in size from 5 to 100 nm 78. A hydrophilic head group and a hydrophobic tail are the two main areas of amphiphiles, also known as surface-active agents (surfactants), which make up micelles 79. The amphiphiles exist as monomers in true solution at low concentrations in an aqueous medium, but when the concentration rises, micelles are generated through aggregation and self-assembly within a limited concentration window 80. The critical micelle concentration (CMC) is the concentration at which micelles develop. Dehydration of the hydrophobic tails creates a favourable state of entropy that drives the creation of micelles over their CMC 81. Furthermore, the hydrophobic polymers can unite and form the micelle core by the creation of Van der Waals linkages. Hydrogen bond networks with the surrounding water are re-established by the resultant hydrophilic shell 82. The CMC of amphiphilic copolymers is typically significantly lower than that of low-molecular-weight surfactants 83. Low molecular weight surfactants typically have a CMC of 10^-3^ to 10^-4^ M, whereas polymeric micelles usually have a CMC of 10-6 to 10-7 M. Polymeric micelles have a longer circulation duration than surfactant micelles because of their low CMC, which keeps them stable at very low polymer concentrations and makes them relatively resistant to dilution 84.

For the purpose of targeting EGFR in lung cancer treatment, Setia *et al*. formulated cetuximab-conjugated redox-sensitive TPGS/HSPC mixed micelles that were loaded with paclitaxel. An approach called dialysis bag diffusion was employed to create the mixed micelles. It was found that the particle size ranged from 162.4 to 239.8 nm and that the surface charge was between -16 mV and -27 mV. After 24 hours, 86% of the drug was released *in vitro* from targeted mixed micelles with a pH of 5.5 and 20 mM GSH. Targeted mixed micelles showed a greater absorption in the 3D A549 tumour spheroid penetration. In addition, after seven days of therapy, the targeted mixed micelles demonstrated a 74% decrease in the growth of the 3D A549 tumour spheroid. Also, the targeted mixed micelles showed more dead cells than the pure drug in the live/dead experiment. The cytotoxicity study also found that targeted mixed micelles were 6.22 times more active than the pure medication. Results from the pharmacokinetic and *in vivo* lung biodistribution investigations were encouraging. Further evaluation of targeting efficiency was conducted in a benzo(a)pyrene-induced lung cancer mice model using imaging modalities such as IVIS and ultrasound/photoacoustic 47.

To target the epidermal growth factor receptor (EGFR) in lung tumour therapy, Setia *et al*. created TPGS-SS nanoparticles (NPs) that contained cetuximab (CTX) coated cabazitaxel (CBZ). Dialysis bag diffusion was used to create the NPs in three different forms: redox-sensitive nontargeted (TPGS-SS-CBZ-NPs), targeted redox-sensitive (CTX-TPGS-SS-CBZ-NPs), and non-redox sensitive nontargeted (TPGS-CBZ-NPs). They evaluated the entrapment effectiveness, surface charge, particle size, polydispersity, and morphology of the NPs that were developed. Furthermore, *in vitro* investigations have been carried out, such as investigations into cellular uptake, drug release, and cytotoxicity. It was discovered that the particle size ranged from 145.6 to 308.06 nm and that the charge over the surface was between -15 and -23 mV. The IC50 values indicates that there is a notable increase in cytotoxicity between formulations compared to CBZ clinical injection. In addition, the A549 cells absorbed considerably more in targeted formulations then pure CM6, according to the in-vitro cellular uptake examination. Additionally, ultrasonic/photoacoustic and intravenous visual imaging were used to evaluate the targeting efficiency of the NPs that were generated 85.

Viswanadh *et al*. formulated nanoparticles based on thiolated TPGS. Conjugating TPGS with 4-amino thiophenol (4-ATP) allowed for the synthesis of a novel redox-sensitive thiolated vitamin E-PEG1000-succinate (TPGH-SH), which was later validated by FTIR and NMR investigations. The following are the results of the size, charge, and percent entrapment screenings conducted on dialysis-prepared, cetuximab-conjugated redox sensitive TPGS-SH nanoparticles (TPGS-SH NP) loaded with docetaxel (DTX). The results showed that the size ranged from 183-227 nm, the surface charge from +18 to +26 mV, and the percent entrapment from 68-71%. Microscopy using SEM, TEM, and AFM has revealed that the NP is uniform in size and has a smooth surface. A drug release of up to 94.5% was noted within 24 hours at pH 5.5 with 20 mM GSH, according to in-vitro release tests conducted in medium containing varying amounts of GSH. Using A549 cells in *in vitro* cytotoxicity, uptake, migration, and apoptotic experiments, the targeted formulation increased cytotoxicity (with a substantially lower IC50 value), uptake, and blocked cell movement. Results from screening CF rats for pharmacokinetic and histological abnormalities were encouraging. Targeted TPGS-SH NP considerably decreased cell quantity compared to the model control, according to *in vivo* effectiveness tests conducted on benzo(a)pyrene-induced lung cancer models in mice 86.

**Figure 6:**
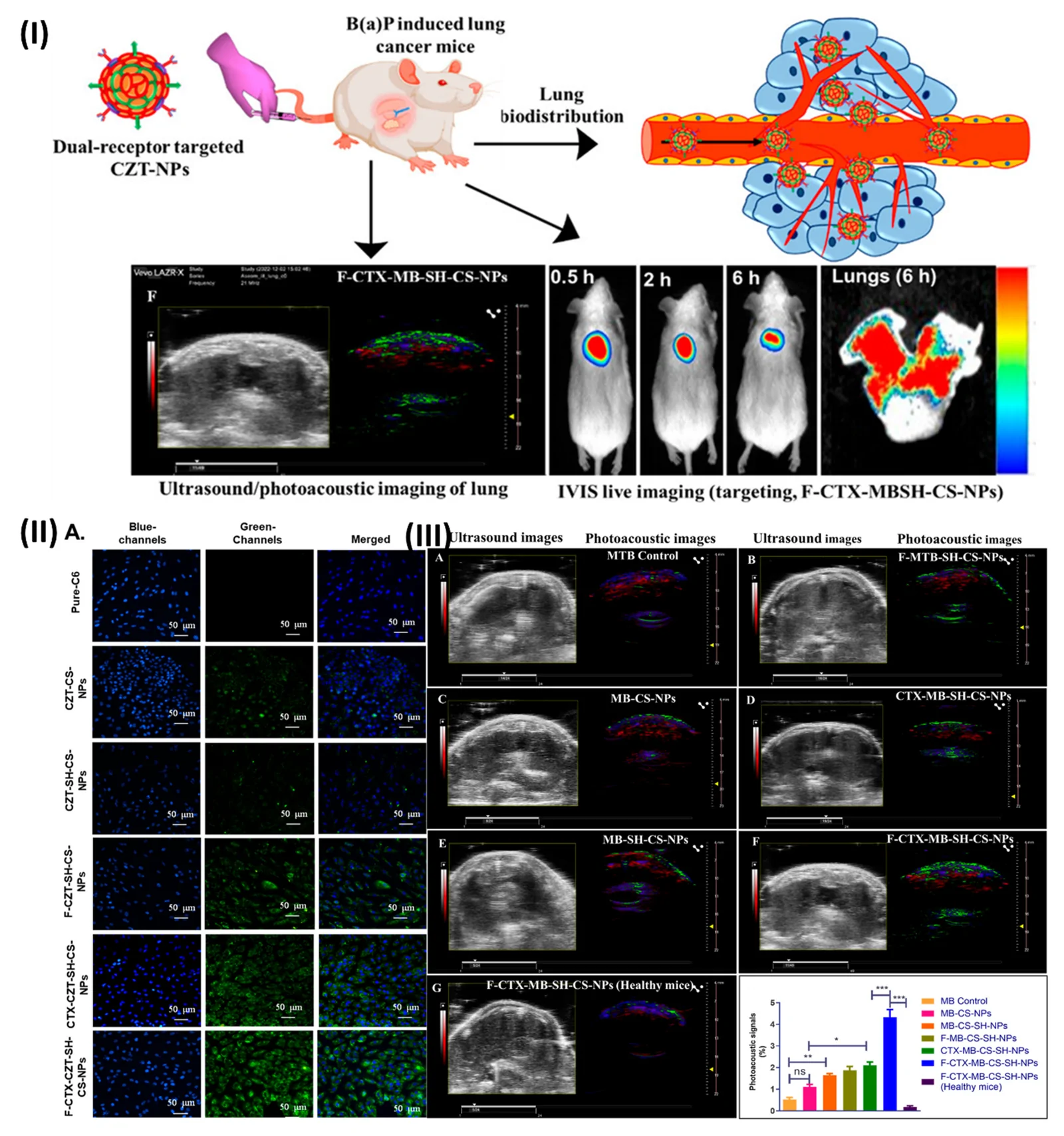
(I) Graphical depiction of nanoparticle delivery to tumor-bearing mice. (II) (A) Cellular uptake of nanoformulations (III) Ultrasound and photoacoustic images of methylene blue and methylene blue loaded nanoformulations (H) Photoacoustic signals of nanoformulation. Reproduced with permission from 87. (ACSPublications©2023).

Vikas *et al*. prepared redox-sensitive tocopheryl polyethylene glycol succinate, and used it to make chitosan nanoparticles loaded with cabazitaxel (CZT) that target two receptors at once. Incorporating synthesised chitosan-folate (F) and TPGS-SH during nanoparticle synthesis and further post-conjugation with cetuximab (CTX) for epidermal growth factor receptor (EGFR) targeting, a combination of pre- and post-conjugation techniques was used to develop dual-receptor targeting redox-sensitive nanoparticles of CZT (F-CTX-CZT-CS-SH-NPs). *In vitro* drug release appeared to be greater in physiological buffer than in redox-sensitive buffer medium (GSH, 20 mM). In contrast to other control nanoparticles, A549 cells exhibited a substantially greater degree of cellular absorption of nanoparticles with multiple targets. In comparison to dual-receptor targeted, non-redox sensitive nanoparticles and CZT clinical injection, F-CTX-CZT-CS-SH-NPs showed a 6.3-fold and 60-fold increase in cytotoxicity, respectively. Additionally, the benzo(a)pyrene lung cancer model showed better anticancer activity with a greater survival rate when treated with F-CTX-CZT-CS-SH-NPs. The novel and redox-sensitive TPGS-SH moiety, the enhanced permeability and retention (EPR) effect of the small-sized nanoparticles, and the dual folate and EGFR mediated augmented endocytosis substantially improved the biodistribution and targeting to the lung, according to investigations employing ultrasound/photoacoustic and IVIS** (Figure 6)** 87.

### 4.3. Targeted nanoparticles for thrombolytic effects

Under physiological or pathological circumstances, thrombus is a localised clotting of blood brought on by an activated coagulation system. As the clinical condition worsens, the blood clot enlarges due to platelet aggregation and subsequently creates partial or total obstruction of the arterial lumen. Early-stage thrombosis may go unnoticed if individuals don't show any symptoms 88. However, a blood flow standstill would result in necrosis of the affected organs and maybe death when blood arteries are almost completely closed. One of the main causes of death worldwide is cerebrovascular illness brought on by thromboembolic vessels 89. Thus, prompt and efficient thrombolysis is desperately needed. Due to its enormous potential to transform medicine by delivering medications to particular parts of the body, targeted drug delivery has been the subject of much research. Targeted medication delivery has demonstrated encouraging treatment results for the most dangerous disorders, including cancer 90. Conventional thrombolytics have limitations that restrict their clinical use, including short half-lives, bleeding issues, and allergic reactions. To improve therapeutic results and reduce side effects, thrombolytics must be delivered precisely to the desired spot. Because haemoglobin, the main molecule in blood that carries oxygen, substantially absorbs laser light at particular wavelengths, PA imaging is especially sensitive to blood flow. This feature allows PA imaging to track blood flow and oxygenation levels in real time 91. Conventional thrombolytics have limitations that restrict their clinical use, including short half-lives, bleeding issues, and allergic reactions. To improve therapeutic results and reduce side effects, thrombolytics must be delivered precisely to the desired spot. Because haemoglobin, the main molecule in blood that carries oxygen, substantially absorbs laser light at particular wavelengths, PA imaging is especially sensitive to blood flow. This feature allows PA imaging to track blood flow and oxygenation levels in real time 92. Because PA imaging is sensitive to vascular changes, it can identify abnormalities in cerebral blood supply and oxygen saturation before major tissue damage occurs. This makes it a useful tool for early stroke diagnosis and prompt management, potentially improving patient outcomes 93.

**Figure 7:**
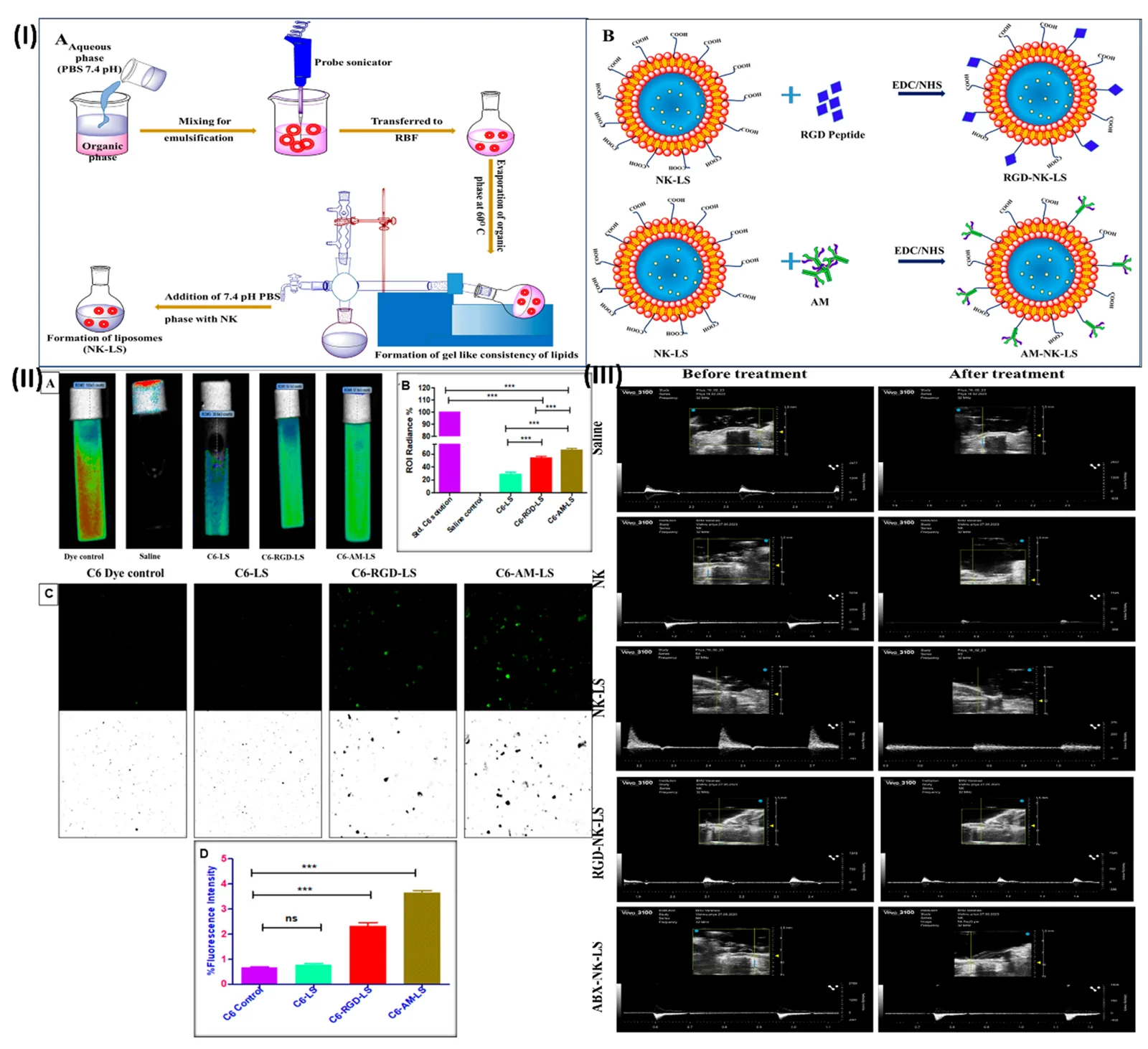
(I) (A) A schematic depicting the NK-LS preparation procedure. (B) Chemistry involving carbodiimides for the conjugation of RGD or AM to NK-LS; (II) A) Bioluminescence images representing the platelet interaction potential of nanoformulations in comparison with the C6 control. B) The bar graph displays the ROI radiance. C) Using C6 dye, CLSM fluorescence and binary pictures of platelet binding affinity, and various formulations D) The relative intensity of fluorescence is shown in a bar graph; (III) Ultrasound imaging. Reproduced with permission from 94. (ACSPublications©2023).

Priya *et al*., formulated nontargeted and targeted liposomes. The targeted liposomes demonstrated a significantly greater (P < 0.05) platelet binding affinity in investigations of platelet interaction. A fibrinolysis investigation employing CLSM imaging and *in vitro* tests using human blood showed that AM-NK-LS was highly effective in preventing blood clots. Additionally, there were no bleeding issues with the targeted liposomes, according to research on clotting time and haemorrhage. In addition, the *in vivo* FeCl3 model demonstrated enhanced thrombolysis and strong targeted liposome affinity for the thrombus site in SD rats. Histopathology and *in vitro* hemocompatibility investigations further confirmed the nanoformulations biocompatibility and safety **(Figure 7)** 94.

In a study, Priya *et al*., formulated abciximab and RGD peptide conjugated rutin loaded liposomes. Liposomes exhibited improved antithrombotic capability in *in vitro* assessments utilising human blood, including blood clot assays, aPTT assays, PT assays, and platelet aggregation analyses. Confocal laser scanning microscopy and *in vitro* imaging were used to assess the efficacy of the clot targeting. The extraordinary ability of targeted liposomes in reducing thrombus formation was demonstrated by a substantial (P < 0.05) increase in their attraction toward activated platelets. In addition, compared to the pure medication, liposomes showed better antithrombotic action in an *in vivo* research conducted on Sprague Dawley rats (FeCl3 model). When given as a liposomal formulation, the bioavailability is higher than when given as RUT, according to the rat pharmacokinetic study. Targeted liposomes outperformed control preparations in an antithrombotic investigation using the tail bleeding assay and clotting time (Swiss Albino mice). The liposomal formulations were also found to be safe for human use and hemocompatible according to an *in vitro* haemolysis study and cytotoxicity assessment. Preparation of liposomal formulations did not cause significant harm in a rat histopathology examination. It follows that RUT encapsulated nontargeted and targeted liposomes showed better antithrombotic potential than RUT and would be a good carrier for future applications 95.

Moreover, Priya *et al*., developed abciximab decorated mesoporous silica nanoparticles (MSN-ABX). Fluorescent images captured in-vitro allowed for an evaluation of the targeted nanoparticles targeting efficiency; these images demonstrated a substantial buildup of nanoparticles coated with ABX towards active platelets. Using an in-vitro imaging technique called photon imager optima, it was confirmed that the DiD dye loaded nanoparticles had a significantly higher affinity for the activated platelets (P < 0.05). Research into the nanoparticle compositions' haemolytic properties showed that they did not break down in normal human blood. Blood clot assays demonstrated that MSN-ABX had better antithrombotic action than clinical injections of ABX, suggesting that it may be a useful vehicle for more precise targeted delivery of ABX *in vitro* 96.

### 4.4. Nanofibers for wound healing

Severe and chronic wounds require extremely complicated cascades of events and pathways to heal. To improve the results of wound healing, a variety of techniques and materials have been investigated 97. Designing nanofibrous mat/wound dressing materials with antibacterial and antibiotic qualities is crucial for accelerating the healing process 98. Compared to traditional dressings, nanofibrous scaffolds provide a number of advantages. They are very porous, feature small pores, and have a high surface area-to-volume ratio. Better exudate absorption, enhanced wound penetration, and the potential to stop more infections are all made possible by these characteristics 99. Electrospun nanofibers could completely change how we treat wounds. To improve the results of wound healing, bioactive substances can be added to these nanofibers, which are composed of both natural and synthetic polymers 100. Researchers can obtain a thorough grasp of how to improve wound healing by investigating the production methods, material choice, and synergistic effects of bioactive chemicals 101.

**Figure 8:**
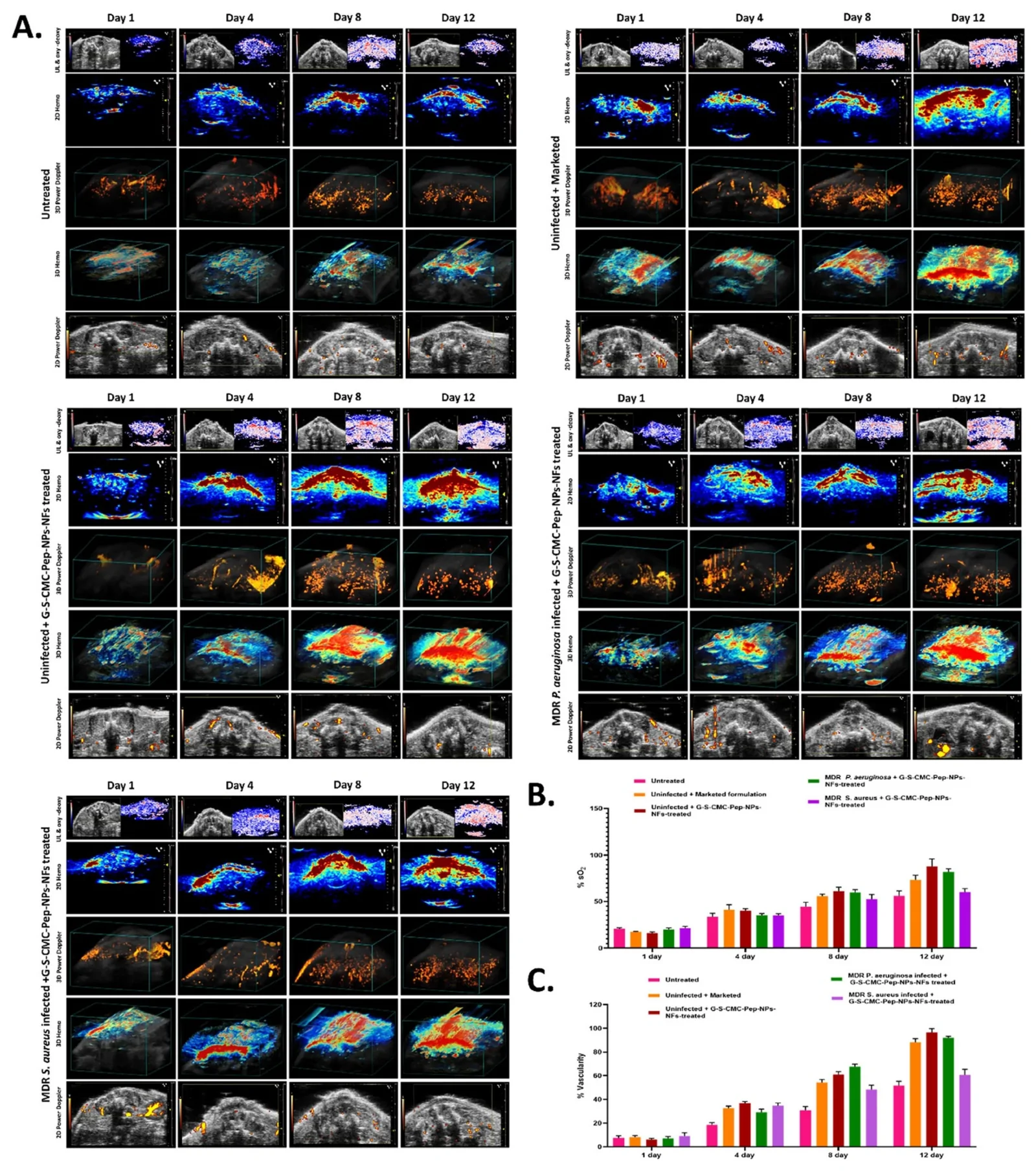
(A) Visualisation of the wounds in each experimental group using oxy-deoxy, power Doppler analysis, and ultrasound/photoacoustic imaging. (B) Superficial oxygen saturation levels in wounds from the respective groups (C) The percentage of wounds with vascularity in each category. Reproduced with permission from 102. (ACSPublications©2025).

Malik *et al*. constructed smart theranostic wound dressings made of gold-silver-LL37 nanoparticles (G-S-CMC-Pep-NPs) coated with carboxymethyl chitosan, which have a surface charge of +34.6 ± 3.7 mV and a size of 155.1 ± 11.2 nm. In addition to showing a maximum zone of inhibition (ZOI) of 21.61 ± 1.06 and 18.85 ± 1.22 mm toward multidrug resistant (MDR) bacteria of P. aeruginosa and S. aureus, respectively, the optimised G-S-CMC-Pep-NPs were found to have minimal inhibitory and bactericidal concentrations in the range of 0.390-0.781 μg/mL. Following a 12-hour treatment, TEM analysis of the microbial cells showed signs of DNA condensation, irregularly undulating and damaged cell walls, and loss of cell wall integrity. Furthermore, when tested on rodent blood, haemolysis experiments showed that G-S-CMC-Pep-NPs had a nonhemolytic profile, demonstrating their exceptional biocompatibility. Additionally, G-S-CMC-Pep-NPs were evenly incorporated into chitosan poly(vinyl alcohol) nanofibers (G-S-CMC-Pep-NPs-NFs) with a size range of 100 to 350 nm. This produced an antimicrobial wound dressing that, when applied to microbially infected wounds in mice, achieved a 92.4% wound closure rate within 12 days of treatment. This study is further supported by *in vivo* optical and ultrasound/photoacoustic imaging, as well as the examination of wound marker protein expression levels. Real-time visualisation, high-resolution spatial imaging, and accurate assessment of blood flow dynamics were made possible by the ultrasound/photoacoustic imaging, which provided a thorough assessment of the intricate wound healing mechanism **(Figure 8)** 102. To improve diabetic wound-healing therapy, Malik *et al*. developed a nanofiber scaffold that was loaded with ZnO nanoparticles generated from trimethyl chitosan (ZnO-TMC-NPs-NFs). The nanoparticle that was created, known as ZnO-TMC-NPs, is 16.1 ± 3.7 nm in size and has a zeta potential of +26.3 ± 1.7 mV. To create a nanoformulation with a size range of 120-240 nm, ZnO-TMC-NPs were integrated into chitosan poly(vinyl-alcohol) nanofiber scaffolds. In addition, the research is strengthened by incorporating *in vivo* optical modalities with sophisticated ultrasound/photoacoustic (PA) imaging, as well as by doing *in vitro* cell line evaluations using L929 mouse fibroblast and A549 lung cancer cell lines. Taken as a whole, the findings highlight the potential of this nanofibrous scaffold as a treatment option for diabetic wound care, especially when combined with TMC-ZnO 103.

## 5. Challenges and clinical translation

Nanotechnology-driven ultrasound/photoacoustic theranostic for multimodal disease applications still confront a number of severe obstacles that prevent their widespread clinical translation, despite notable breakthroughs. The poor targeting efficacy of nanoparticles due to their quick clearance, nonspecific biodistribution, and biological barriers that prevent aggregation at sick tissues is one of the main challenges 104. Important biosafety issues are also raised by the long-term toxicity, poor biodegradability, immunogenicity, and possible bioaccumulation of inorganic nanomaterials. Imaging, drug delivery, photothermal therapy, and biosensing can all be integrated into a single nanoplatform, however doing so requires complicated manufacturing processes, low reproducibility, and expensive production. Moreover, therapeutic efficacy may be jeopardised by early drug leakage, physiologically unstable nanoparticles, and shorter circulation times. Ultrasound and photoacoustic signals frequently undergo attenuation and dispersion in deep tissues, which limits their penetration depth and lowers their imaging sensitivity. Additionally, endogenous chromophores like melanin and haemoglobin may affect the specificity of photoacoustic contrast 105.

In addition, research into molecular probes or targeted contrast agents that can amplify PAI signals in pathological tissues or biomarkers should be prioritised 106. It is possible to improve early diagnosis, treatment planning, and therapy response monitoring with the incorporation of molecular imaging capabilities into PAI, which in turn allows for the non-invasive identification of molecular targets. The potential for biomolecular targeting methods, theranostic agents, and nanoparticle-based contrast agents to improve the specificity and sensitivity of PAI in molecular imaging applications is substantial 107. But nanoparticles as contrast agents in human clinical trials have not materialised yet. Exogenous agent use in humans is fraught with difficulty due to the lengthy and stringent regulatory licensing process that is necessary for this use. However, nanoparticles have shown promise in PAI in large-scale preclinical studies, highlighting their powers in contrast-enhanced imaging, tailored medication delivery, and therapy monitoring. Although it is tough to bring PA medicines based on nanoparticles to human clinical trials, these agents show promise for expanding PAI's capabilities in the areas of clinical diagnosis and theranostic applications 108.

The FDA offers various pathways for product approval; however, a De Novo or Premarket Approval (PMA) pathway is required for PAI due to the lack of a predicate device. There are still very few PAI systems that have received clinical approval 109. It wasn't until 2021 that Seno Medical Instruments Inc.'s ImagioTM Breast Imaging System was granted the first ever pre-market approval (PMA) by the FDA in the US. As mentioned earlier, the report's convincing data showed that PAI could greatly improve the specificity of traditional ultrasound-based breast cancer diagnosis, which was the basis for the approval. Furthermore, in 2019 the MSOT Acuity (PAI solely) and in 2021 the MSOT Acuity Echo (dual-modal PAI and USI) PAI systems from iThera Medical GmbH were CE marked 110. The clinical translation of PAI was significantly advanced by these accomplishments. Through ongoing cooperation and coordinated efforts among several disciplines, PAI has the ability to revolutionise medical diagnostics and enhance patient outcomes in various clinical settings.

Included in the PMA application were results from clinical studies and bench tests conducted using bespoke phantoms, as well as results from modelling and testing conducted in accordance with current IEEE standards for ultrasonic systems. Dimensionality, co-registration, accuracy, precision, out-of-plane optical absorption effects, sensitivity, linearity, dynamic range (SNR and contrast-to-noise ratio), depth detection, and elevational and spatial resolutions were all included in the phantom study's conclusions. Prior to the supplementary approval in June of 2022, the FDA had already approved the PMA in January of 2021. As is customary for 510(k) submissions using USI systems, additional data regarding the USI mode of testing was also included 111.

It is difficult to show that an existing diagnostic workflow may be significantly improved upon. Radiologists have access to a variety of imaging modalities, and diagnostic breast imaging produces large volumes. Complementing pre-existing modalities (mammography, ultrasound, and MRI), PAI provides additional information that may seem complementary or contradicting 112. Typically, radiologists will search for questionable signs in the clinical workflow and then conduct a biopsy to confirm them. If radiologists want to boost specificity without sacrificing sensitivity, they need to be confident when using PAI's extra information to lower suspicion. To aid radiologists in image interpretation, a decision support tool (DST) was created for the Imagio system. The DST begins by using the user's score for ultrasound features, photoacoustic features, BI-RADS data, and other clinical data, such as age, lesion size, and depth. Ultrasound features include things like peripheral and boundary zones, tissue shape, internal texture, and sound transmission. Photoacoustic features include things like internal vessels, internal Hb, and internal blush. The DST then uses the results of the AI models to provide a cancer risk assessment. As far as regulators are concerned, the DST is an integral component of the device. While the Medical Device Directive has been in place to approve medical devices in the EU, the new Medical Device Regulation went into effect in 2021, creating new hurdles for manufacturers looking to sell their wares in the EU market and adding more red tape to the process. The transitional period for high-risk devices has been extended to 2027 and for lower-risk devices to 2028. During this time, manufacturers have the option to submit data for approval under the Medical Device Directive 113.

## 6. Conclusion

The hybrid imaging approach known as PA imaging may examine specific chromophores at tissue depths as little as three to five centimetres, producing high-resolution images. The biggest challenge with PA method is getting light to penetrate deep into tissue. Due to their lengthy photostability and high optical absorption, nanoparticles could serve as contrast agents and produce powerful ultrasonic signals, allowing them to circumvent this obstacle. Image reconstruction, sensing, and, under some circumstances, therapeutic applications all make use of these signals. PAT has great promise as an imaging modality with several potential uses in the biomedical field. Non-invasively visualising anatomical features for clinical applications like cancer prognosis and haemodynamic information for functional imaging with high spatiotemporal resolution is made possible with label-free PAT by using endogenous biomolecules like haemoglobin, DNA/RNA, lipid, melanin, and water as the PA signal generating compound. Active targeting exogenous contrast chemicals are necessary to enable targeted and disease-targeting imaging, which is essential for PAT to reach its full potential. This supplementary skill is critical for tracking the development of disease, determining the efficacy of treatment, and identifying a wide variety of malignancies at various stages. Exogenous contrast compounds have the potential to be utilised as treatment agents for PTT/PDT in the future as well. Synergies between endogenous and external contrasts can be achieved, however, because PAT can use different wavelengths of light to image several contrasts at once. The biocompatibility of exogenous contrast agents and PAT systems with respect to water solubility, cytotoxicity, and renal clearance is a key concern when it comes to their introduction to the clinical context. This is even though PA contrast agents, including exogenous substances, have demonstrated potential for use in deep-tissue imaging. Additional research and development are necessary to successfully transition PAI into routine clinical use, despite its impressive potential in multiple therapeutic applications. For PAI to reach its full potential as an effective clinical imaging modality, there must be technological advancements, protocol standardisation, validation through clinical trials, and the creation of specific contrast agents. In addition, forming nanoparticles that are both photostable and biocompatible will completely change the landscape of mPA technology, opening the door to its widespread use in the clinic in the not-too-distant future. We have covered numerous nanoparticles used in PA imaging, sensing, and spectroscopic methods in this article. Not only does this study shed light on imaging, but it also describes numerous therapeutic, theranostics, and drug delivery uses of nanoparticles in PA approaches. This paper is valuable for researchers in numerous domains because it covers the latest and broad application areas of nanoparticle-based PA algorithms.
